# Zebrafish as a Translational Model: An Experimental Alternative to Study the Mechanisms Involved in Anosmia and Possible Neurodegenerative Aspects of COVID-19?

**DOI:** 10.1523/ENEURO.0027-21.2021

**Published:** 2021-05-28

**Authors:** Karla C. M. Costa, Tamires A. V. Brigante, Gabriel G. Fernandes, Davi S. Scomparin, Franciele F. Scarante, Danielle P. de Oliveira, Alline C. Campos

**Affiliations:** 1 Pharmacology of Neuroplasticity Laboratory, Department of Pharmacology, Ribeirão Preto Medical School, University of São Paulo, São Paulo, Brazil, 14049-900,; 2 EcoHumanTox Laboratory, Department of Clinical, Toxicological and Bromatological Analysis, School of Pharmaceutical Science of Ribeirão Preto, University of São Paulo, São Paulo, Brazil 14049-900

**Keywords:** anosmia, encephalitis, olfactory system, pandemic, SARS-CoV-2, zebrafish

## Abstract

The Coronavirus disease-2019 (COVID-19) presents a variability of clinical symptoms, ranging from asymptomatic to severe respiratory and systemic conditions. In a cohort of patients, the Severe Acute Respiratory Syndrome Coronavirus (SARS-CoV-2), beyond the classical respiratory manifestations, induces anosmia. Evidence has suggested SARS-CoV-2-induced anosmia can be the result of neurodegeneration of the olfactory pathway. Neurologic symptoms associated with COVID-19 have been reported; however, the precise mechanism and possible long-lasting effects remain poorly investigated. Preclinical models are valuable tools for describing and testing new possible treatments for neurologic disorders. In this way, the zebrafish (*Danio rerio*) organism model represents an attractive tool in the field of neuroscience, showing economic and logistic advantages besides genetic and physiologic similarities with mammalian, including the brain structure and functions. Besides, its external embryonic development, high availability of eggs, and fast development allows easy genetic manipulation and fast replications. In the present review, we suggest that the zebrafish model can be advantageous to investigate the neurologic features of COVID-19.

## Visual Abstract

**Figure:**
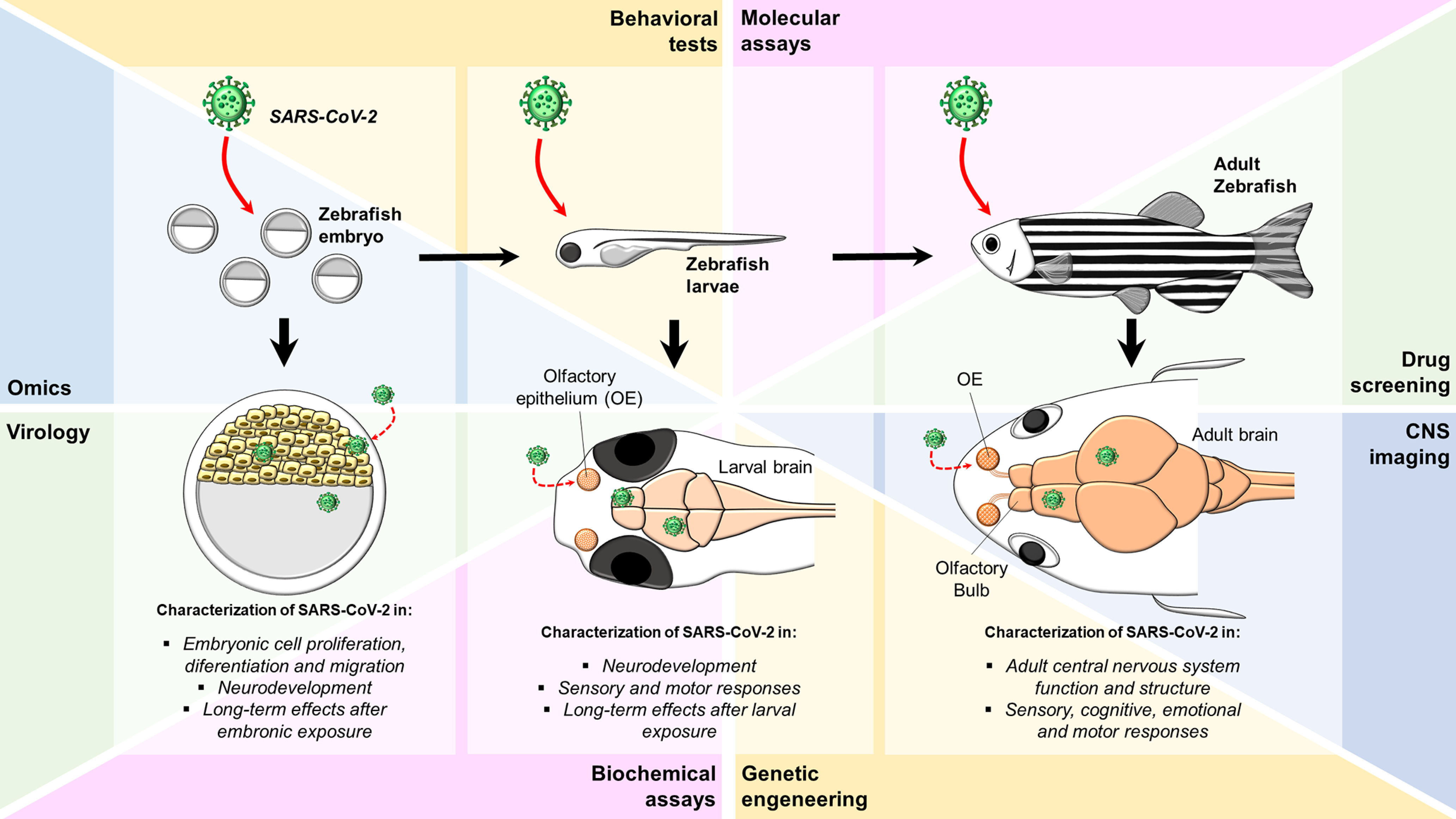

## Significance Statement

The Coronavirus disease-2019 (COVID-19) is characterized not only by respiratory, but also neurologic symptoms that may persevere even longer than the respiratory condition. However, some questions remain unanswered. How does the Severe Acute Respiratory Syndrome Coronavirus 2 (SARS-CoV-2) enter the brain? Which brain regions are infected? How does the virus affect our behavioral and sensorial responses? Can the brain infection induce alterations that last longer? We propose that the zebrafish is a suitable model to study the effects of SARS-CoV-2 in the brain, especially because of the similarities between the fish’ and the human brain. Zebrafish is a cheap animal model and several reproducible and fast animal tests can be used to investigate its behavioral and sensory functions.

## COVID-19 and Society: The Urgent Need of a Rapid and Fast Animal Model

The novel strain of coronavirus responsible for the Coronavirus disease-2019 (COVID-19) emerged in December 2019 in Wuhan, a province in China (Rothan and Byrareddy, 2020). In a short period, COVID-19 cases have rapidly spread worldwide causing frightful rates of morbidity and mortality (Jain et al., 2020) and declared as a global public health threat by the World Health Organization in March 2020 (World Health Organization, 2020).

Because of efforts made by different groups of scientists, the etiologic agent of this new pandemic was characterized as a β coronavirus named Severe Acute Respiratory Syndrome-Coronavirus 2 (SARS-CoV-2), which is closely related to its previous relative SARS-CoV, responsible for the SARS outbreak in China during the early 2000s (Ksiazek et al., 2003). Similar to its “older cousin,” spike proteins S1 and S2 of the SARS-CoV-2 use the host angiotensin-converting enzyme 2 (ACE2) as a receptor (Walls et al., 2020) to initiate its entrance into the cell. This interaction with ACE2 seems to be primed by a proteolytic cleavage of the spike (S) protein by the transmembrane protease serine (TMPRSS2), responsible for the virus interaction with its target receptor (Hoffmann et al., 2020; Ou et al., 2020). Moreover, unlike SARS-CoV, the invasive mechanism of SARS-CoV-2 seems to involve preactivation by furin, a proprotein convertase that reduces the dependence of the novel coronavirus on recruiting proteases of the target cells for its successful entry (Shang et al., 2020).

Early epidemiological studies suggested that most patients infected with SARS-CoV-2 developed none or mild symptoms, similar to common flu, caused by influenza viruses, such as fever, cough, fatigue, rhinorrhea, sneezing, and sore throat. However, recent evidences showed that beyond the respiratory system infection, SARS-CoV-2 could also produce a severe syndrome with its collection of symptoms: severe pneumonia, important damage in the cardiovascular system, including thrombosis, persistent anosmia, and in some quite often neurologic symptoms (encephalitis, disturbed consciousness, and cerebrovascular accident; Duong et al., 2020; Ellul et al., 2020; Mao et al., 2020; Rothan and Byrareddy, 2020; Wu and McGoogan, 2020; Xu et al., 2020).

A great number of patients with COVID-19 have described neurologic complications associated with the viral infection (Helms et al., 2020; Mao et al., 2020). These case reports raise questions regarding the SARS-CoV-2 neurotropism, and how it contributes to the postinfection complications in the CNS (Netland et al., 2008; Huang et al., 2020; Zhang, 2020). A recent study (Chen et al., 2021) showed that ACE2, the target for SARS-CoV-2 entrance, could be identified in different components of CNS such as neurons, astrocytes, and oligodendrocytes. Many brain structures exhibited a high expression of ACE2 including the olfactory bulb, a region that has been associated with anosmia (loss of smell sense), a recurrent symptom also reported by patients with COVID-19 (Chen et al., 2021). Besides, other neurologic manifestations have already been identified in patients diagnosed with COVID-19, such as headache, confusion, and disabling strokes (Iadecola et al., 2020). However, it is not well characterized how the SARS-CoV-2 affects directly the CNS or to what extent the neurologic disorders are consequences to secondary mechanisms.

The ACE2 is an enzyme expressed in many tissues, including the brain cells (Gheblawi et al., 2020), which can infer that once in the brain parenchyma, SARS-CoV-2 could be neuroinvasive. Additionally, SARS-CoV-2 is mainly transmitted through expelled virus-laden droplets, which can be inhaled by another person, leaving the virus exposed mainly to epithelium-like tissues throughout the respiratory tract. In this way, recent evidence suggests that the virus may enter the brain via the olfactory system through the nasal cavity, affecting breath control (Li et al., 2020a). Although other species of virus are capable of penetrating the CNS (for detailed review, see Koyuncu et al., 2013) the precise mechanism involved in SARS-CoV-2 neurologic manifestation remains poorly understood.

Other viruses from the coronavirus family have been shown to use the olfactory pathway to enter the brain following intranasal inoculation. Perlman et al. (1990) showed that the *Mouse hepatitis virus* (MHV), a neurotropic coronavirus, is detected in brain areas neuroanatomically connected to the olfactory nerve after intranasal exposure to the virus. Furthermore, surgical bulbectomy prevented the entry of MHV into the brain via the olfactory pathway (Perlman et al., 1990). Moreover, in transgenic mice that express the human ACE2 receptor under the control of the human cytokeratin 18 (K18) promoter, SARS-CoV, after intranasal inoculation, was rapidly found in the olfactory bulb and in brain regions that have first or second-order connections with the olfactory bulb, indicating that the virus enters the brain through the olfactory tract and reaches connected brain areas through transneuronal spreading (Netland et al., 2008). A similar mechanism of neuro-propagation from the olfactory bulb to neuroanatomically associated areas was described for the human coronavirus strain OC43 (Dubé et al., 2018).

If SARS-CoV-2 uses the olfactory route to enter the brain and migrates through transsynaptic spreading to brain regions directly or indirectly connected to the olfactory bulb, the virus can potentially invade the thalamus, the hypothalamus, cortical regions, the midbrain, and even the brainstem. This could be related not only to the neurologic manifestations described in several COVID-19 patients but could also contribute to the respiratory symptoms since the neuro-invasiveness of SARS-CoV-2 could compromise the respiratory center of the brainstem (Gandhi et al., 2020; Li et al., 2020b; Manganelli et al., 2020; Dey et al., 2021).

The assumption of the olfactory pathway as the route of entry for SARS-CoV-2 into the brain is supported by the expression of ACE2 in the olfactory bulb and in brain regions that are directly connected to the olfactory tract (Chen et al., 2021). The ACE2 receptor is expressed in numerous cell types, including excitatory and inhibitory neurons, as well as microglia, oligodendrocytes, oligodendrocyte-precursor cells, and astrocytes (Chen et al., 2021). Moreover, TMPRSS2, a protease that participates in the cellular transfection of the virion, and neuropilin-1, a signaling molecule shown to increase SARS-CoV-2 infectivity, are also expressed in the olfactory neuroepithelium (Butowt and Bilinska, 2020; Matschke et al., 2020; Karuppan et al., 2021).

Histopathological evidence also supports that SARS-CoV-2 could directly infect cells from the olfactory tract. Meinhardt et al. (2021) reported the presence of the SARS-CoV-2 spike (S) protein within neuronal cells in the olfactory mucosa from patients with COVID-19. They also found SARS-CoV-2 RNA in the olfactory bulb and in brain regions that receive projections from the olfactory tract (Meinhardt et al., 2021). In K18-hACE2 mice, after intranasal inoculation of SARS-CoV-2, the virus was detected in the brain tissue on day 3 after infection. By day 6 after the inoculation, SARS-CoV-2 was detectable in the olfactory bulb, cortex, cerebellum, and hippocampus of K18-hACE2 mice (Kumari et al., 2021).

The SARS-CoV-2 infection has been associated with brain damage in the olfactory bulb and its neuroanatomically connected areas. Stoyanov et al. (2020) described severe neurodegeneration and inflammatory cell infiltration in the olfactory bulb of two COVID-19 patients. In a postmortem case series conducted in Germany, neuropathological analysis of glial activation patterns revealed a high degree of astrogliosis and microgliosis in the olfactory bulb, with low levels of cytotoxic-T cell infiltration (Matschke et al., 2020). The study also reported activation of microglial cells and infiltration of CD8-positive lymphocytes in the brainstem and a level of astrogliosis in the frontal cortex of COVID-19 patients (Matschke et al., 2020). In K18-hACE mice, SARS-CoV-2 intranasal inoculation triggered an increase in proinflammatory cytokines and chemokines in the brain and induced neuronal cell death in the hippocampus, cortex, and cerebellum (Kumari et al., 2021).

Animal models are the founding steps toward a better understanding of biological processes. Rodents-based models, such as mice and rats are widely used in biomedical research, but because of their intrinsic phenotype, their use in COVID-19 is limited. This restriction is associated with crucial amino acid variations in the primary structure of the ACE2 receptor (Chan et al., 2020). To circumvent this limitation, transgenic mice models expressing human ACE2 (hACE2) are available, but there are some limitations regarding their use: (1) there are reports that SARS-CoV-2 induces mortality on 7 d postinfection (dpi) limiting their use on long-term experiments; (2) because of the high demand of research labs, their breeding and distribution are limited. Other animal models are being studied, such as ferrets (Kim et al., 2020), golden hamster (Chan et al., 2020; Sia et al., 2020), and non-human primates (Bao et al., 2020; Rockx et al., 2020), but to perform experiments using these animals, several laboratories must adapt their animal housing facilities, and some of them take several weeks to produce offspring (for detailed review of animal models available, see Johansen et al., 2020).

Since there is an urgency to establish new and effective experimental models to understand the neurologic components associated with COVID-19, our goal with this review is to present evidences supporting the use of *Danio rerio*, commonly known as zebrafish, as a powerful tool to comprehend to which extent the SARS-CoV-2 may affect and alter the CNS homeostasis.

### Zebrafish as a rapid and replicable experimental model

Zebrafish (*D. rerio*) is a small teleost that originated from the South of Asia and is characterized by the blue-black longitudinal stripes alternating with silver-white stripes that extend across its body (Lawrence, 2007). After fertilization, a large number of eggs (∼100 per day) is generated. The eggs are transparent, making it possible to observe the structures of the zebrafish’s organs during development. They achieve the larval stage from 2 to 3 d after the fertilization period in which the organogenesis of many structures is still not complete (Kimmel et al., 1995; Spence et al., 2007). Its small size, easy maintenance in the laboratory, external fertilization, transparency of the embryos, and well-defined stages of development make the zebrafish an attractive model for research in many fields (Kimmel et al., 1995; Howe et al., 2013; Diniz et al., 2015; Kundap et al., 2017; Saad et al., 2017; Ayala-Nunez et al., 2019). Besides, zebrafish have genetics and physiologic similarities with mammals, increasing their value as a powerful tool on the bench side of the scientific methods (MacRae and Peterson, 2015).

Genetic sequences in zebrafish have been studied since the 1980s. Njølstad et al. (1988) characterized a zebrafish *homeobox* and found that this sequence shares similarities that lead to the same encoded proteins as the murine domain *hox-2.1*. In the following years, other zebrafish genes were sequenced and compared with mammalian. Several genes showed similarities in the sequence, expression, and encoded proteins (Njølstad et al., 1988; Krauss et al., 1991). Postlethwait et al. (1998) mapped 144 zebrafish genes and described large conservation of chromosome segments between zebrafish and mammalian, including some human genes. Sahly et al. (1999) described the expression of eya1 that is involved in embryogenesis, and their results predicted a protein with 84.7% similarities with its human orthologue. Blaker-Lee et al. (2012) described zebrafish genes orthologues with human genes involved in brain disorders.

Howe et al. (2013) accomplished the significant step to evaluate the homology between human and zebrafish genomes in 2013. The zebrafish genome comprises around 26 thousand protein-coding genes in 26 pairs of chromosomes, while the human genome is composed of between 20–25 thousand genes in 23 pairs of chromosomes (Howe et al., 2013; International Human Genome Sequencing Consortium, 2001). The researchers observed that >70% of human genes have an orthologue in zebrafish. The authors also found that some human genes have no orthologue in zebrafish, even presenting related receptors. These facts indicate that zebrafish have proteins with similar functions to human proteins; however, they encoded for different genes in humans and zebrafish. They also compared the zebrafish genes with the human genes described in the Online Mendelian Inheritance in Man (OMIM) database, which catalog genes involved in morbidity development. More than 80% of the 3176 genes described have an orthologue in zebrafish, allowing the use of zebrafish to investigate numerous human morbidities (Howe et al., 2013).

Over the years, many authors described genes associated with CNS development (Mack-Bucher et al., 2007; Xu et al., 2011; Blaker-Lee et al., 2012; Umans et al., 2017; Wu et al., 2019), CNS functions (Cocco et al., 2017; Laboissonniere et al., 2018), neurogenesis (Krauss et al., 1991; Byrd and Brunjes, 2001; GermanÀ et al., 2011), suggesting that zebrafish is a suitable model to evaluate neural disturbance mechanisms, efficacy, and toxicology of potential treatments. Besides, it is extremely important to highlight that although both species, human and zebrafish, possess a distinct systemic respiratory mechanism, studies have shown that the zebrafish swimming bladder (a specialized organ responsible for a proper buoyancy of teleosts) possesses anatomic, embryological, and transcriptome resemblances with human (and other mammals) lungs (Zheng et al., 2011). Moreover, Jonz and Nurse (2003) showed that the neuroepithelial cells (NECs) which are involved in respiratory control in mammals, are also present in larval and adult zebrafish. As indicated by immunolabeling, the NECs have vesicles containing the neurotransmitter serotonin, which seems to play a role in the respiratory system (Jonz and Nurse, 2003). These aspects suggest that not only the neuroinvasion could be studied (as discussed below) but also other systemic COVID-19s manifestation and complication.

Moreover, using zebrafish is a tremendous advantage because it has been accepted as an alternative model since it fulfills the 3R’s principles: reduction, refinement, and replacement. The current European Union legislation (European Parliament, 2010) on the protection of animals used for scientific purposes considers that the early stages of the development of zebrafish [until 5 d postfertilization (dpf)] do not need protection since the procedures conducted will not provide suffering. The period before embryos and larvae reach exogenous feeding is used as criteria by EU Directive 2010/63/EU to define the period that fish do not require regulation (Scholz et al., 2008; Beekhuijzen et al., 2015; Bhusnure et al., 2015). Using zebrafish for research purposes also contributes to reduction because it allows the economy of animals of higher orders in many research steps, such as screening of substances in drug discovery. Also, because it undergoes the processes of absorption, distribution, metabolism, and excretion, zebrafish’s use reduces the difference in results obtained between *in vitro* and *in vivo* tests (Bhusnure et al., 2015).

### Zebrafish’s susceptibility to SARS-CoV-2 infection

In addition to the receptor ACE2, the TMPRSS2, the proteases cathepsin L, trypsin, and furin were referred to mediate the virus’ entry into the cells. Between the countless human-related proteins that zebrafish presents, there is also evidence of these enzymes involved in SARS-CoV-2 infection. This information can be used to design experiments that could help understand the virus infection’s whole process or even study potential drugs, which target these enzymes.

Cathepsin L is present in zebrafish since the early stage of development. Expression of the gene *catL* can be detected even around 7 h postfertilization (hpf), and the expression is higher at the hatching time. Cathepsin L is involved in the development and hatching processes (Vogel and Gerster, 1997). Dana et al. (2019) used zebrafish as the *in vivo* model to ensure the efficacy of a selective probe synthesized to study human cathepsin L. Therefore, cathepsin L was proposed as a biomarker for chemical exposure by evaluating its enzymatic activity in zebrafish embryos (Küster, 2005).

Trypsin and its receptor *Par2* are present in zebrafish. The receptor *Par2* is ubiquitously distributed while the trypsin was found in higher concentrations in the gill, nasal cavity, and mouth of embryo-larvae stages of zebrafish. It seems to play a role in response to injury, protecting the fish from bleeding (Kim et al., 2009; Xu et al., 2011).

Expression of furin genes was also described in zebrafish even in early stages and seemed to be relevant in the development and immunologic system (Walker et al., 2006; Ojanen et al., 2015).

The protease TMPRSS2 has only been studied in zebrafish in its rearranged form TMPRSS2-ERG, a biomarker of prostate cancer. However, there is still evidence that zebrafish have an orthologue to the TMPRSS2 protease (Bansal et al., 2014; Butler et al., 2017). Ohler and Becker-Pauly (2011) observed high evolutionary conservation between the genes encoding TMPRSS4 in zebrafish and humans. They also demonstrated that the early development of zebrafish was affected by the TMPRSS4 gene silencing (Ohler and Becker-Pauly, 2011).

The zebrafish ACE2 receptor region that interacts with the protein S seems to share 50–64% similarities with the same human region (Kraus et al., 2020). This characteristic was explored in a recent preprint paper, in which the authors showed that an exposure of zebrafish larvae to SARS-CoV-2 protein S receptor-binding domain (RBD) induced augmentation on heart rate at 5 and 7 dpf. This parameter was normalized after larvae exposure to captopril, suggesting the participation of the ACE2 receptor on this cardiovascular parameter (Kraus et al., 2020). Intriguingly, the authors also observed that intranasal delivery to RBD resulted in disrupted integrity of the olfactory system, such as edema, hemorrhage, and apical loss of olfactory sensory neurons, accompanied by reduced electro-olfactogram signal (Kraus et al., 2020).

### Modeling zebrafish’s viral infection: a powerful tool to understand SARS-CoV-2

The zebrafish organism is gaining considerable visibility as an animal model to study pathogenesis and to screen new potential compounds for the treatment of different bacterial (Takaki et al., 2013) and viral infections (Gabor et al., 2014) that also affects the respiratory system in humans. Zebrafish has several components of human immunity. The innate immunity is present since the larvae hatched from the egg, whereas adaptive immunity takes weeks to fully develop (Lam et al., 2004; Novoa and Figueras, 2012). Despite the evolutionary distance, there are several similarities between the human and zebrafish immune systems; those include cellular components, i.e., macrophages, neutrophils, eosinophils, dendritic cells, and signal transduction pathways to eliminate the intruders (Goody et al., 2014).

The innate immune system first recognizes viral infections through mechanisms that may rely on toll-like receptors (TLRs), among them 3, 7, 8, and 9, retinoic acid-inducible gene I-like receptors, nucleotide oligomerization domain-like receptors, and receptors that detect DNA in the cytoplasm (for more details, see Thompson and Iwasaki, 2008). Although the exact mechanism responsible for an immune response toward SARS-CoV-2 is still under loose, it is conceivable to assume that it interacts with one or more of these receptors. Elucidating this piece of the puzzle may help comprehend one of the significant complications concerning the COVID-19’s evolution and mortality.

It is assumed that the exacerbated immune response toward SARS-CoV-2 elicits a colossal increase in peripheral proinflammatory cytokine and chemokine that ultimately can promote organ failure and death, an effect known as a “cytokine storm” (Mehta et al., 2020; Qin et al., 2020). In this way and assuming the importance of generating new rapid models to study this phenomenon in the laboratory, Kraus et al. (2020) have established zebrafish, in different stages of life, as useful models to investigate the pathophysiological effects of SARS-CoV-2 infection on both olfactory and cardiovascular system. The study revealed that the immune response of zebrafish to recombinant protein S parallel those observed in humans that present a mild-form of COVID-19. More specifically, the authors observed an increase in mRNA of antiviral response and proinflammatory cytokines, such as interleukin-1β (IL-1β), tumor necrosis-α (TNFα), interleukin-17 (IL-17), and chemokine ligand 20 (CCL20; Kraus et al., 2020).

Highlighting the converge immune response of zebrafish and humans, Progatzky et al. (2019) elegantly showed that mimicking viral infection on the upper respiratory tract through intranasal administration of resiquimod (TLR 7/8 agonist) promoted an increase in proinflammatory cytokines expressions, such as IL-1β, type-I interferons (IFN- a crucial antiviral signaling molecule) and TNF-α. The study provided insights that zebrafish responses to resiquimod stimuli were similar to those observed in humans, both presented an augmentation of TNF-α and IFN-γ in a time-dependent manner, while in mice the levels of TNF-α drastically reduced below basal levels after 1 h. Moreover, the authors also observed that the exposure to polyinosinic:polycytidylic acid (poly (I:C), a viral double-strand RNA mimetic) produced an upregulation of genes responsible for TNF-α, IL-6, and IFN- γ only in mice, while there was no observable changes neither on humans or zebrafish after 1 or 8 h exposure (Progatzky et al., 2019). More intriguing, Gabor et al. (2014) described zebrafish as a valuable animal model to investigate the infection of different strains of human *Influenza A virus* (IAV), which similarly to SARS-CoV-2, is characterized as a causative agent of respiratory disease. The infected animals presented an increase in viral burden, recapitulating the immune and clinical symptoms of influenza infections in humans, showing an increased and sustained IFN expression, edema, and tissue destruction with multiple organ involvement. Besides, the treatment of infected zebrafish with Zanamivir, an anti-influenza compound, was able to reduced mortality and the expression of viral gene product, demonstrating the validity of zebrafish as a model to screen new antiviral compounds for IAV treatment (Gabor et al., 2014).

Besides its use on cytokine profile to immune challenges, zebrafish can be genetically manipulated to gain or loss-of-function of a myriad of proteins and may help to characterize the SARS-CoV-2 kinetic and dynamic. These have been applied to understand *in vivo* viral infection kinetic, cell tropism, phagocytic behavior, spatiotemporal activation of antiviral pathways (Ding et al., 2011; Palha et al., 2013; Passoni et al., 2017). In this direction, Ding et al. (2011) investigated whether zebrafish could be a potential model for *Hepatitis C virus* (HCV) replication research. The animals microinjected with *NS5B*-plasmids demonstrated viral particles amplified in the liver. Even without any abnormalities in the development of zebrafish larvae, the viral amplification induced a similar gene expression pattern as those observed in human hepatocytes (increased expression of *Argsyn*, *Hsp70*, *Leugpcr*, *ScarF2*, *Rasgbd*, and *chemokine-1* genes). Beyond the immune similarities, the administration of anti-HCV drugs, ribavirin and oxymatrine, was able to reduce the amplification rate in infected larvae, demonstrating again the great potential of this model as a tool for HCV drug screening (Ding et al., 2011). Another suitable example of zebrafish versatility to study viral infections was demonstrated by Passoni et al. (2017) using a transgenic zebrafish and genetically modified *Chikungunya virus* (CHIKV) and *Sinbdis virus* (SINV), both pathogens that sometimes are associated with neuropathies. The study showed that microinjection of green fluorescent protein (GFP)-expressing CHIKV and SINV on zebrafish larvae promoted an increase in GFP intensity in different organs. The authors also observed that SINV possesses a broad organ tropism as showed by GFP expression on central and peripheral organs after 1 d postinjection. Moreover, this study has demonstrated, by using a transgenic zebrafish expressing the red fluorescent protein (RFP) on endothelial cells, that only GFP-CHIKV colocalized with RFP-endothelium after 1 and 2 d postinfection, revealing that CHIKV, but not SINV, infects endothelial cells of the blood-brain barrier (BBB). Besides, they also studied the “Trojan-horse” neuroinvasion hypothesis, a secondary mechanism for CNS entry. By using a transgenic zebrafish line in which macrophages (including the microglia) express *mCherry*, a red cytosolic fluorescent protein, the authors showed that neither CHIKV nor SINV use this alternative route for CNS infection. Altogether, these results aid by unveiling possible tools to understand viruses neuroinvasion mechanism on humans (Passoni et al., 2017).

## The Zebrafish CNS, Olfactory Pathway, and Its Possible Relevance in the Context of COVID-19

Among the preclinical models applied o to study the physiological and abnormal brain functions, rodents still are the most employed animals in the neuroscience field. However, other animal models, like zebrafish, are emerging as promisor candidates (Keifer and Summers, 2016).

**Figure 1.**
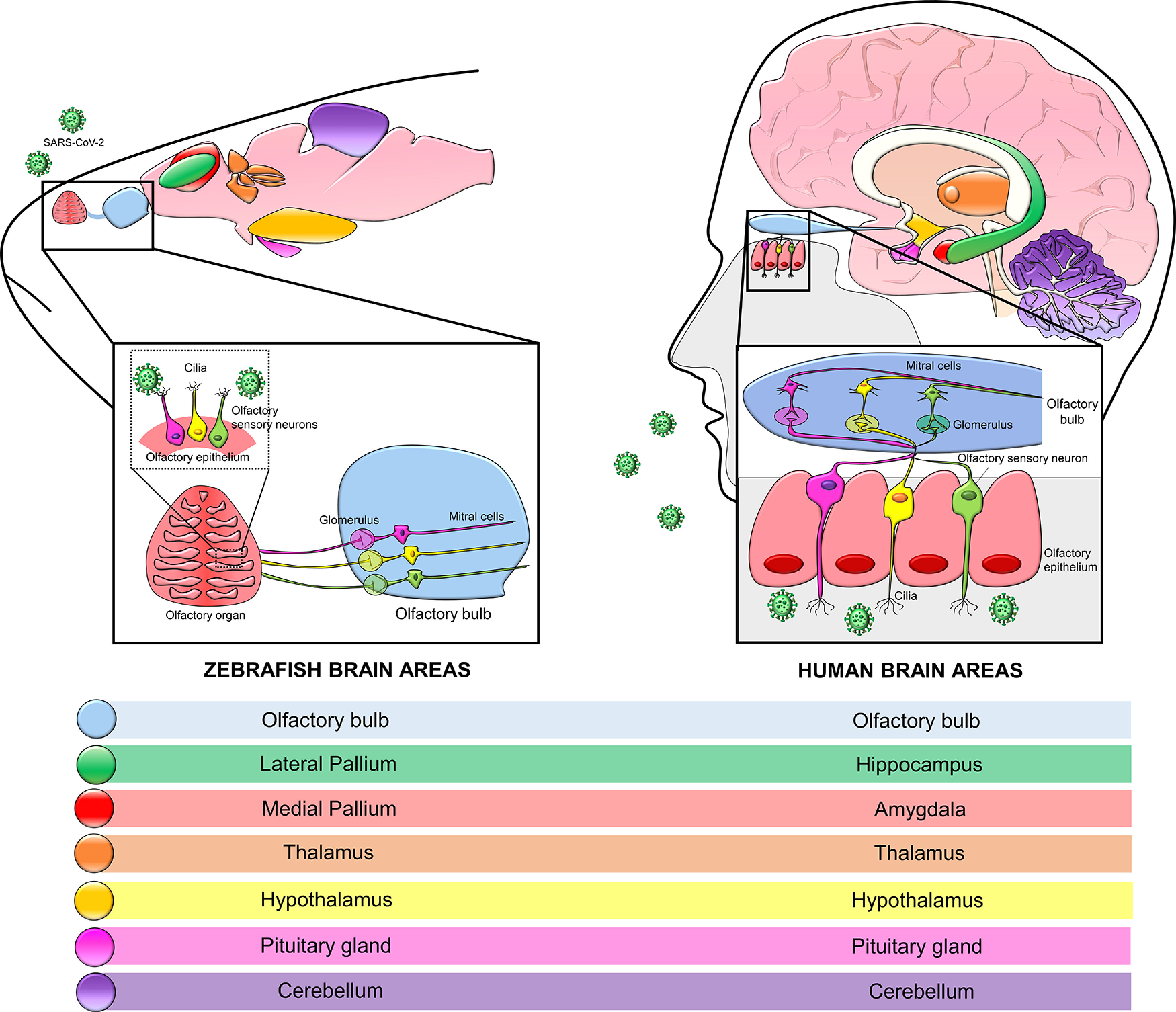
Zebrafish versus human olfactory system as one of the possible routes for the entrance of SARS-CoV-2 into the central nervous system. The human olfactory neuroepithelium comprehends ACE2-expressing olfactory sensory neurons that communicate with mitral cells in the olfactory bulb. The structure and cytoarchitecture of zebrafish’s olfactory neuroepithelia and olfactory bulb are similar to that found in humans. Moreover, human brain areas present functional and structural correlates in the zebrafish brain.

As exemplified in Figure 1, the zebrafish’s brains share many neuroarchitecture and cellular morphology with mammals, including humans, characteristics that configure it as a valuable tool to model a wide range of human brain disorders (Kalueff et al., 2014; Stewart et al., 2014). Moreover, zebrafish have a very similar neurotransmitter system when compared with humans. Among the already neurotransmitters and neuromodulators characterized in this teleost are the GABA, glutamate, dopamine, noradrenaline, serotonin, acetylcholine, and histamine systems, which can already be observed at the early stages of zebrafish embryo and seems to play similar roles to those observed on mammals (for detailed review, see Horzmann and Freeman, 2016).

Regarding anatomy and physiology, despite its smaller cerebral hemispheres, the zebrafish brain presents a highly conserved organization, keeping important similarities when compared with the human brain. For instance, zebrafish have all typical sensory functions (vision, olfaction, taste, hearing, and tactile), and of note, all the sensory pathways share significant homology with humans (Byrd et al., 1996; Wullimann and Puelles, 1999). Highlighting similarities with humans’ brain organization, the BBB is present on zebrafish, which develops on 3 dpf, and controls small molecules’ permeability (Fleming et al., 2013).

Like mammals, the adult zebrafish brain is divided into the forebrain, midbrain, and hindbrain. The forebrain is the anterior part and includes the telencephalon and diencephalon. The midbrain is the portion between the forebrain and hindbrain, divided into optic tectum, torus semicircularis, torus longitudinalis, and midbrain tegmentum. Besides, the posterior portion of the brain is the hindbrain, which is composed of the cerebellum and medulla oblongata (Wullimann et al., 1996; Mueller et al., 2011; Folgueira et al., 2012). Functional similarities are also observed, for instance, the lateral pallium of zebrafish contains homologous structures to the hippocampus (Fig. 1), a structure that is derived from the medial pallium and is closely related to spatial memory (Eichenbaum et al., 1999; Rodríguez et al., 2002). Besides, the medial pallium of zebrafish presents an amygdaloid-like function (Fig. 1), a brain structure involved in the aversive learning in humans (Martín et al., 2011; Mueller et al., 2011; von Trotha et al., 2014). Similar to the olfactory system of mammals, neurons in the olfactory bulb of zebrafish project toward the telencephalon and diencephalon (Wullimann and Puelles, 1999), suggesting that the zebrafish present an anatomic and functional organization that parallel those observed on humans (Fig. 1). In this sense, Miyasaka et al. (2014) used single-neuron labeling analysis in larvae zebrafish to reveal that axons at glomerular clusters in the olfactory bulb target the regions of the forebrain such as the posterior telencephalon, ventral telencephalon, the right habenula, the posterior tuberculum and the olfactory bulb itself in ipsilateral and contralateral regions. Noteworthy in the context of COVID-19, the olfactory system of zebrafish also shares many similarities with those observed in terrestrial mammals, including humans (Hildebrand and Shepherd, 1997; Howe et al., 2013; Saraiva et al., 2015; Fig. 1).

In zebrafish, the olfactory system can be divided into two main structures: the peripheral organ that organizes itself in a pair of rosettes on the nasal cavity, and the superior structure that is represented by the olfactory bulb and attached structures, responsible for diverse behavioral responses (Baier and Korsching, 1994; Korsching et al., 1997; Friedrich et al., 2013). The odorant receptors, responsible for the primary signal transduction in the periphery organ, follow the one neuron – one receptor rule, by which the sensory neurons can express only one receptor of many existing types. These neurons expressing the same receptors converge their axons onto a specific location in the olfactory bulb, and different odors can activate different types of compromised neurons, encoding a specific pattern of activation responsible for singular odors sensations (Barth et al., 1996; Friedrich and Korsching, 1997).

Another advantage is that zebrafish’s olfactory system has a quick development detecting and discriminating odors between 2 and 4 dpf (Lindsay, 2004; Vitebsky et al., 2005). Moreover, this teleost also presents anatomically olfactory components more accessible than other vertebrates, allowing invasive manipulations with an increased rate of survival (Baier and Korsching, 1994; Iqbal and Byrd-Jacobs, 2010; Paskin and Byrd-Jacobs, 2012; White et al., 2015). Altogether, these characteristics allow a quick and effective investigation of SARS-CoV-2 infection both on embryonic and adult olfactory systems.

### Zebrafish as a model of neurologic disorders

How SARS-CoV-2 encroaches on the CNS is a question that remains unexplained. As mentioned, the primary hypothesis is that the virus can reach the neuronal cells through the olfactory nerves, promoting complications related to the olfactory perception, such as anosmia (for detailed review, see Lao et al., 2020). Corroborating this premise, functional analysis using magnetic resonance imaging of a patient who suffered from COVID-19 anosmia and dysgeusia suggested an altered cortical activity that normalized after resolving these symptoms (Politi et al., 2020).

Another hypothesis suggests that SARS-CoV-2 can promote an entry by a hematogenous route, either infecting the BBB cells or camouflaging itself in immune cells to enter the central nervous system as Trojan horses, both leading to possible neuroinflammatory boards. This hypothesis is based on the neurovascular symptoms, such as stroke, that affect many contaminated patients (Kumar et al., 2020). These possible pathways to SARS-CoV-2 entry in the CNS agree with the secondary neurologic symptoms presented by the patients, which can range from simple headaches to hemorrhagic strokes (Berger, 2020; Orrù et al., 2020), a serious condition that can increase the risk of death (for more detailed review of SARS-CoV-2 neuroinvasion, see Iadecola et al., 2020).

The zebrafish model shows up great features once it is already used as a neurologic model for the disorders studies because of its similarities with the human CNS and the easy access to the communications routes among the brain areas, mainly in the embryonic phase (MacRae and Peterson, 2003). The neurovascular system can be evaluated in zebrafish and robust protocols and neuroimages assays that can easily show the dimension of the stroke-induced (Crilly et al., 2019) and can lead to a neuroinflammatory correlation process, as happens in parallel in SARS-CoV-2 infection. Furthermore, transsynaptic tracing using viruses to enable gene transfer has been used in zebrafish to study neuronal connection (Mundell et al., 2015). In this sense, similar techniques in addition to fluorescent labeling could be used to evaluate the spreading of SARS-CoV-2 in the CNS. Beyond that, the virus’ pathways to entry in the CNS can be easily verified in the zebrafish model, as already happened with *Chikungunya virus* and *Sindbis virus* through fluorescent techniques (Passoni et al., 2017), a protocol that may be applied in COVID-19 research.

More than evaluating the acute consequences of the infection and its correlation with the CNS, zebrafish can provide long-term results about these consequences. It is unknown whether and how the interaction with SARS-CoV-2 and the brain can lead to the worsening of other preexistent neurologic conditions or precipitate the appearance of symptoms in people susceptible to neurologic diseases in a long-term manner, either by the possible capacity of the virus to cause brain injury directly or through affecting the people by promoting a stressful overload because of pandemic era events, like social distancing.

Anosmia and ageusia, symptoms experience for most patients infected with SARS-CoV-2, are also reported in the initial stages of neurodegenerative diseases such as Alzheimer’s and Parkinson’s disease (Tarakad and Jankovic, 2017; Marin et al., 2018). Therefore, the concern that the new coronavirus can promote a certain degree of neurodegeneration, or even modulate the CNS homeostasis to lead to a new pathologic state, as happened with one patient that presents a quick development of Parkinson’s disease starting after SARS-CoV-2 diagnosis (Cohen et al., 2020). To evaluate whether these factors can induce long-term changes, the zebrafish is a valuable tool since it is an established model to study neurodegeneration even in the context of neurodegenerative disorders (Tierney, 2011; Martín-Jiménez et al., 2015), and can be used to assess infection course and neurodegeneration relationship in a longitudinal way, because of quick and easy mode to obtain young to aged zebrafish (Kao et al., 2014). Teleosts also present an interesting great feature that can serve as an advantage against the rodent models: the possibility to regenerate the brain tissue (Kizil et al., 2012; Gemberling et al., 2013).

People who do not suffer from SARS-CoV-2 infection can experiment with its effect on their brains, even if not directly. Many studies reveal that pandemic issues tend to increase mood disorders (such as depression and anxiety) diagnosis worldwide (Tang et al., 2021), a problem that may have worse consequences in the future. One manner of studying these consequences is through behavioral analysis, an area where zebrafish can get great importance once it demonstrates a wide range of complex behaviors that can be evaluated and recently gained notoriety (Jesuthasan, 2012). Moreover, software that analyzes many behavioral types (such as ANY-maze software for rodents) can be found for zebrafish (Khan et al., 2017), which adds to its short development and great reproducibility and can save even more time and make searching even easier.

## Evaluation of SARS-CoV-2 Neurologic Alterations through Zebrafish’s Behavior

Behavioral tests have been used in neuroscience, neuropharmacology, and neurotoxicity fields to access numerous CNS disorders. This tool can help to elucidate the mechanisms involved in pathologies or injuries caused by chemical exposure and may be conducted with adults (Abreu et al., 2017; Kundap et al., 2017; Genario et al., 2020; Müller et al., 2020) or larvae zebrafish (Vitebsky et al., 2005; Krishnan et al., 2014; Ko et al., 2019), and may use the exploratory pattern to stimuli, such as light vibration and odorants exposure (Kalueff et al., 2013). In this context, many protocols have been developed and cataloged over the past decade; for instance, Kalueff et al. (2013) clustered 190 distinct behavioral phenotypes that include anxiety-like, freeze, spasm behaviors, and seizures.

In the light of COVID-19 CNS alterations, anosmia may be evaluated in zebrafish at the behavioral level. In this direction, assessing locomotor activity with adults or larvae can be used to evaluate the olfactory system function (Krishnan et al., 2014; Abreu et al., 2017). It has been described that anosmia induced by lidocaine and ZnSO4 promoted anxiety-like behaviors in zebrafish (Abreu et al., 2016, 2017). Also, protocols aiming at evaluating the olfaction capacity can include predator responses. These stimuli can be produced by using the skin of the predator as stimuli (Volz et al., 2020), skin injury mixture to evoke stress-related behavior (Diaz-Verdugo et al., 2019), or water-soluble chemicals such as food preparation, for the search for food behavior (Braubach et al., 2009; Chen et al., 2019), amino acids (Krishnan et al., 2014; Wakisaka et al., 2017), or bile acids (Koide et al., 2009; Wakisaka et al., 2017). Interestingly, even in the first stages of development, it is possible to evaluate zebrafish’s motor activity. At almost 24 hpf (19–27 hpf), zebrafish have spontaneously tail coiling measured by frequency and duration of occurrence. These assays enable the evaluation of SARS-CoV-2 infection during development and may shed light on possible future alterations (Menelaou et al., 2008; Selderslaghs et al., 2013).

Regarding long-term behavioral alterations promoted by SARS-CoV-2 that may suggest neurodegeneration, several reports are evaluating the zebrafish memory performance in a distinct context such as spatial memory function (Williams et al., 2002), stress-induced memory impairment (Gaikwad et al., 2011), and scopolamine-induced memory impairment (Richetti et al., 2011).

### To model zebrafish into a preclinical and efficient model of COVID-19 model: exploring the strategies and tools

The generation of the first transgenic mammal in the 1980s opened a wide venue for this field (Gordon and Ruddle, 1981). Nowadays, genetic manipulation of animals is an invaluable tool for understanding human disease. Although almost 40 years have passed since Palmiter et al. (1992) successfully generated “gigantic” mice, the steps used to generate transgenic mice are almost the same, which involve: (1) constructing a transgene; (2) obtaining fertilized eggs from female mice donors; (3) microinjections of the transgene material into the pronuclei/zygote; (4) implanting the modified zygote into pseudo-pregnant female mice; (5) genotype the transgene offspring. These several steps and the limitation of offspring generated by each pregnancy lead to several months of work until a viable transgenic mouse is established. These limitations and the urgency of viable tools to understand the behavior of SARS-CoV-2 during this pandemic raise the undeniable necessity of a rapid and reliable animal model.

Zebrafish’s genetic manipulation offers an undeniable resource. The first transgenic zebrafish were obtained in the 1980s (Stuart et al., 1988) that showed that DNA transplantation into the zebrafish’s embryo could be integrated and inherited by their germline. Almost a decade later, two distinct groups successfully created a transgenic zebrafish that expressed green fluorescent protein (GFP) under promoter-control that conveyed a tissue-dependent expression pattern (Long et al., 1997; Higashijima, 2008). These rudimentary protocols highlight the advantages of zebrafish over rodents. The former produces hundreds of eggs that can be fertilized and generated offspring’s in a shorter period; their eggs are translucent, allowing *in vivo* imaging studies during the development of the CNS (Long et al., 1997).

Nowadays, several techniques have been described to generate transgenic zebrafish, such as knock-down with morpholinos, insertion of exogenous DNA with *Tol2* transposons, knock-in for fate-mapping studies (Kizil et al., 2013; Kesavan et al., 2017; Rafferty and Quinn, 2018). To provide organized information regarding the strains of zebrafish, plasmids, and antibodies accessible, online database centers were created, such as the Zebrafish Information Network (Varshney et al., 2016; Gut et al., 2017).

### Real-time visualization of the zebrafish’s CNS and SARS-CoV-2

Imaging tools can be very useful for understanding the possible alterations triggered in the CNS, facilitating, and improving clinical prognosis. Beyond the conventional immunohistochemistry protocols, new strategies allowed *in vivo* screening and whole-brain assays in the zebrafish. These tools can be very important in the characterization of the neuroinvasion promoted by SARS-CoV-2 (Solomon et al., 2020).

Some fluorescent markers, like the GFP and the yellow fluorescent protein (YFP), can be used to label neuronal projections during zebrafish neurodevelopment. Indeed, the combined expression of different fluorescent markers refers to the transgenic technology known as Brainbow, in which several neurons can be individually labeled through genetic recombination (Cai et al., 2013). This technology was recently applied to the zebrafish model (Pan et al., 2011), giving rise to the successful transgenic tool named Zebrabow (Pan et al., 2013), enabling the broad study of the zebrafish’s CNS.

Moreover, the functional analyses and activity of neurons can be achieved through genomic tools like the genetically-encoded calcium indicators (Ahrens et al., 2013) and the optogenetic neuromodulation (Knafo and Wyart, 2015). Both strategies can be easily inserted in the zebrafish genome by driving the expression of many protein-based systems for genome engineering (Kawakami et al., 2004; Scott et al., 2007), targeting a specific neuronal population of interest. Unlike other vertebrate models, using light-sheet microscopy, the whole-brain activity of larval zebrafish can be rapidly obtained without attaching any fiber optic cables (Ahrens et al., 2013).

In addition to the techniques for functional analyses in the brain of zebrafish larvae, there are the fluorescent false neurotransmitters that are capable of measuring the *in vivo* uptake and release of a specific neurotransmitter. These synapse-markers are based on a fluorescent label of the synaptic vesicle or the extracellular neurotransmitters (Gubernator et al., 2009).

Because of the lack of transparency and a larger brain, imaging the CNS of adult zebrafish is a challenge. However, methods like the use of different solvents to remove the excess of lipids and reduce light scattering (Ertürk et al., 2012), allow transforming a fixed and opaque organ into a transparent one, enabling an *in situ* analysis of the whole adult brain (Susaki et al., 2014). Another recent protocol applied for imaging the brain of adult zebrafish is the contrast-enhanced X-ray micro-computed tomography, which provides a 3D brain visualization (Babaei et al., 2016). Furthermore, the optical coherence tomography enables an *in vivo* and non-invasive real-time 3D imaging of the adult zebrafish brain anatomy (Rao et al., 2009).

### Molecular and biochemical analysis in the zebrafish model

As in whole science, molecular biology techniques are essential in virology research, even more, if we consider that viruses do not have macro features to be analyzed, which gives great importance to genomic and proteomic analysis. Despite being a relatively new model, zebrafish can supply the molecular research with optimized protocols that ensure not only technical quality but also relative simplicity, presenting several examples of its use in viral infection research in zebrafish (Goody et al., 2014).

The Western blotting (WB) technique is one of the most used techniques to evaluate protein content and can be performed to verify proteins involved with viral infection as well as the proteins that lead the immune response to the viral infection. Furthermore, zebrafish provide a broad spectrum of possibilities once the protein content can be measured and compared in different developmental phases with relative speed. Newly published protocols also guarantee sample economy and optimized protein extraction of a single zebrafish larvae or embryo for WB, which added to the high fertility of zebrafish culminates in a large amount of sample in short periods (Schnabel et al., 2019). Besides that, the availability of antibodies specific to zebrafish samples is increasing.

Despite the utility of WB to provide previously known protein quantity information, much more unknown proteins can be related to viral infections and disease progression, further in novel viral infections such as COVID-19. In this case, zebrafish can be a useful tool in the research as well, which can involve specifically protocols for proteomics assays that can lead to a protein screening in diverse scenarios, from electrophoresis to the mass spectrometry techniques, both in adults and embryos (Link et al., 2006; Singh et al., 2013).

If the aim of the work concentrates on the genomic profile, zebrafish can help mainly in the evaluation of viral infection kinetics studies and genome expression measurements, not only for viral particles or whole-genome but also for the genes of target-proteins for viruses (Goody et al., 2014). The evaluation of the RNA content can be a powerful tool against SARS-CoV-2, once this virus has a single-stranded RNA, enabling the investigation of a plausible connection between the viral proteins and the ACE2 target-protein in humans. Furthermore, using the reverse transcription-PCR quantitative real-time (RT-qPCR) technique, ACE2 domains in different neurons and regions in zebrafish were already identified (Chou et al., 2006).

Besides the aforementioned, biochemical assays comprehend the understanding of the chemical reactions that occur inside the organism. This type of essay allows us to elucidate the mechanism of action of many substances. Many procedures to perform the biochemical assays developed for other species (Ellman et al., 1961; Misra and Fridovich, 1972; Bradford, 1976; Aebi, 1984; Draper and Hadley, 1990; LeBel et al., 1992; Frasco and Guilhermino, 2002) has been adapted and used in zebrafish with great results (Jin et al., 2015; Abe et al., 2018; Agostini et al., 2018; Maharajan et al., 2018; Sarasamma et al., 2018, 2019; Bernardo et al., 2019; Mocelin et al., 2019; Schmitt et al., 2019; Ünal et al., 2019; Walpitagama et al., 2019).

Different enzymes or processes can be studied by biochemical assays in zebrafish and may be used to understand the possible alterations promoted by SARS-CoV-2 at the cellular and tissue level. For instance, some tests allow the determination of the reactive oxygen species, the lipid peroxidation, and the activity of the enzymes involved in antioxidant mechanisms, such as superoxide dismutase, catalase, and glutathione-S-transferase (Diniz et al., 2015; Jin et al., 2015; Sarasamma et al., 2018). Besides, some assays enable the measurement of distinct neurotransmitters activity, such as the choline, GABA, glutamate, glycine, dopamine, and 5-hydroxytryptamine (Abe et al., 2018; Agostini et al., 2018; Sarasamma et al., 2018, 2019; Schmitt et al., 2019; Walpitagama et al., 2019).

As was previously discussed, some enzymes are involved in SARS-CoV-2 entrance into the cells, such as the proteases cathepsin L, TMRPSS, trypsin, and furin. These enzymes could be used as a target for new drug development. A methodology to evaluate cathepsin L activity was already developed and tested in zebrafish (Dana et al., 2019). It was developed for different purposes, happened before SARS-CoV-2 was brought to light, but it could be helpful to assess the efficacy of drugs that target cathepsin L.

### The “omics-era” and zebrafish

Much of our knowledge about single cells evolved drastically within the past decades with advanced molecular biology techniques. Nowadays, it is possible to comprehend the system-behavior in a single-cell resolution (Wagner and Klein, 2020).

The advent of omics analysis, which refers to an integrative discipline with the objective of mapping genes and proteins and comprehends the interactions and relationship among them and the environment surrounding the cell, has enabled the in-depth knowledge of the different systems and how diseases may disturb the homeostasis of these systems (Jehan, 2019; Unterman et al., 2020).

Regarding COVID-19, the omics approach has already been used to clarify some of the molecular alterations promoted by SARS-CoV-2 infection in humans (Song et al., 2020; Unterman et al., 2020). For instance, lipidomes analysis revealed that some plasma metabolites differed from COVID-19 patients compared with healthy controls (Song et al., 2020), and a multiomics single-cells analysis revealed that worse response to SARS-CoV-2 infection was associated with predominant interferon type-1 response across different immune cells accompanied with an imbalance between innate and adaptive immunity (Unterman et al., 2020). However, preclinical studies using the omics approach and SARS-CoV-2 dynamics still need to be explored.

In this direction, some shreds of evidence strongly suggest that zebrafish omics may be used to screen the biological activity of distinct chemical compounds, testing its teratogenicity and toxicity (Sukardi et al., 2010; Hung et al., 2012). Also, the proteomics approach has already been used to understand age-related neurodegenerative diseases, such as Alzheimer’s and Parkinson’s (Abramsson et al., 2010; Mushtaq et al., 2013), alterations on the Rett syndrome model (Cortelazzo et al., 2017), to demonstrate the similarities between humans and zebrafish plasma composition (Li et al., 2016) and neutrophils (Singh et al., 2013), to explore different biomarkers for liver dysfunction (Ayobahan et al., 2020), to explore the dynamics of extracellular matrix composition during heart regeneration (Garcia-Puig et al., 2019), and to investigate the alterations promoted by spring viremia of *Carp virus* in the zebrafish (Liu et al., 2020). These pieces of evidence highlight that omics tools may be used for different purposes on the zebrafish model and are a powerful tool for a better understanding of human diseases with distinct etiology, including COVID-19.

## Conclusion

Among all the animal models, zebrafish has proven to be an essential and powerful tool for human disease analysis. General attributes, like the low-cost maintenance, external fertilization, a large number of eggs, rapid life cycle, transparency of the embryos, and the several available techniques make this vertebrate an attractive model for research. Zebrafish also display genetics and physiologic similarities with mammalian, including the brain structure and functions, which highlights its great potential as an animal model for studying the neurologic components associated with SARS-CoV-2 infection. Several of the genetic, behavioral, cellular, molecular, and biochemical approaches already standardized in other animal models are also applicable in this teleost. Altogether, those advantages ensure reliable, fast, and reproducible results for COVID-19 analysis when compared with other animal models. The goal of proposing an innovative animal model like zebrafish to study neurologic components related to SARS-CoV-2 infection is ambitious but will be of great value for more robust and effective analysis.

### Search strategy

All the relevant articles were identified and collected from the PubMed database from April 1, 2020, up to November 30, 2020. The following search terms were used (alone or in combination): zebrafish, SARS-CoV-2, COVID-19, CNS, encephalitis, neurologic disorders, anosmia, ciliopathy, olfactory system, olfactory bulb, olfactory neurons, ACE2, TMPRSS2, immunity, behavior, genetic manipulation, imaging tools, molecular analysis, biochemical analysis, omics. The reference list was generated based on papers relevant to the topics discussed in this review.

## Synthesis

Reviewing Editor: Eran Perlson, Tel Aviv University Decisions are customarily a result of the Reviewing Editor and the peer reviewers coming together and discussing their recommendations until a consensus is reached. When revisions are invited, a fact-based synthesis statement explaining their decision and outlining what is needed to prepare a revision will be listed below. The following reviewer(s) agreed to reveal their identity: Jennifer Freeman.

This review discusses the use of zebrafish to study effects and mechanisms of COVID-associated anosmia and neurodegeneration. It is well written and potentially interesting. There are some aspects that should be better addressed:

- Differences between respiratory modes in human and fishes sould be considered in terms of adaptive mechanisms and how do they impact traslation in the context of COVID. Suggestions: https://doi.org/10.1371/journal.pone.0024019

- Some of the most important supporting references are from repositories (biorxiv) and not peer-reviewed yet. While this makes sense given recency, it would be good to provide some additional support from published papers. For example, zebrafish has been used for studying pulmonary diseases and neurodegeneration (see for instance https://doi.org/10.1016/j.nbd.2014.08.004) and also models of tubercolisis used for drug-screeing (https://doi.org/10.1038/nprot.2013.068). Also it will benefit the readers to add more details on studies that have specifically used the zebrafish to investigate viral outcomes

- One major pathway for neurodegenerative complications associated to COVID is transynaptic spreading from the olfactory epithelium down to brain regions (or more specifically to respiratory centers). Here it may be useful to discuss how the zebrafish can help to understand this process. Certainly, knowledge about the connectome at single cell resolution is worh mentionning (see https://doi.org/10.1038/ncomms4639)
